# Baicalein suppresses adhesion and biofilm formation in *Candida auris*

**DOI:** 10.1128/spectrum.01097-26

**Published:** 2026-08-04

**Authors:** Can Li, Hui Wu, Yemei Wang, Wenfan Wei, Daqiang Wu, Xin Feng, Tianming Wang, Changzhong Wang

**Affiliations:** 1 Department of Pathogenic Biology and Immunology, College of Integrated Chinese and Western Medicine (College of Life Science), Anhui University of Chinese Medicine 665596 , Hefei, China; 2 Institute of Integrated Traditional Chinese and Western Medicine, Anhui University of Chinese Medicine 117843, Hefei, China; Stony Brook University, Stony Brook, New York, USA

**Keywords:** *Candida auris*, baicalein, adhesion, biofilms

## Abstract

*Candida auris*, an emerging multidrug-resistant fungal pathogen, poses a severe global public health threat owing to its high nosocomial transmissibility, considerable mortality, and widespread antifungal resistance. Baicalein (BE), a major bioactive constituent of *Scutellaria baicalensis* Georgi, exhibits notable antifungal potential, yet its specific molecular mechanisms against *C. auris* remain poorly elucidated. In this study, we determined the antifungal activity of BE against multiple *C. auris* isolates, assessed its effects on fungal growth, virulence attributes, adhesion, and biofilm development, validated its *in vivo* protective efficacy in a *Galleria mellonella* infection model, and explored the underlying mechanisms via transcriptomic sequencing. BE exerted potent and consistent antifungal activity against all tested strains, with a minimum inhibitory concentration of 1 μg/mL, minimum fungicidal concentrations of 4–8 μg/mL, and a sessile minimum inhibitory concentration of 32 μg/mL. It time- and concentration-dependently suppressed fungal growth, virulence factor expression, adhesion to biological and non-biological surfaces, and biofilm formation, while conferring significant *in vivo* protection against *C. auris* infection. Transcriptomic analysis identified 11 adhesion- and biofilm-associated target genes, among which *SCF1* and *ALS1* served as pivotal regulators. Collectively, BE exerts robust anti*-C*. *auris* effects by modulating key target gene expression to interfere with multiple virulence-related processes, providing experimental support for its development as a novel antifungal agent for *C. auris* infection treatment.

**IMPORTANCE:**

The emerging multidrug-resistant fungal pathogen *Candida auris* has become a critical global public health concern. Its pronounced nosocomial transmissibility, high infection-associated mortality, and extensive cross-resistance to mainstream antifungal agents have created substantial unmet needs in clinical treatment and nosocomial infection control. In this study, we systematically validated the *in vitro* and *in vivo* antifungal activity of baicalein against *C. auris* and elucidated the molecular mechanism underlying its modulation of virulence-related genes. Our findings provide a pivotal experimental basis for the development of novel antifungal therapeutics targeting *C. auris* infections.

## INTRODUCTION

*Candida auris* was first isolated and identified in Japan in 2009 (1). As an emerging multidrug-resistant fungal pathogen, it has been designated as a “super fungus” in the academic circles, a designation primarily attributed to its high propensity for inducing nosocomial infection outbreaks, a mortality rate ranging from 30% to 72% (2–4), and a broad spectrum of antifungal resistance. This species exhibits remarkable genetic diversity and can be categorized into six distinct evolutionary lineages. Within more than a decade following its initial isolation and identification, its geographic range of infection has rapidly expanded to more than 50 countries and regions across six continents globally (5). The prevalence of the COVID-19 pandemic has exacerbated its transmission dynamics, thereby posing a severe and urgent threat to global public health security (6). In 2019, the Centers for Disease Control and Prevention incorporated it into the list of urgent threat pathogens associated with antimicrobial resistance (7); in 2022, the World Health Organization classified it into the critical priority group in the first-ever released “Fungal Priority Pathogen List” (8).

*C. auris* possesses extremely potent adhesive and colonizing capacities, allowing it to persist for extended periods on human skin and the surfaces of inanimate objects, a trait that consequently facilitates contamination of the clinical environment (9). Notably, immunocompromised patients, inpatients with long-term indwelling invasive catheters, and susceptible nosocomial populations exhibit a significantly elevated risk of skin colonization and bloodstream infection. Skin colonization serves not only as an independent risk factor for secondary invasive bloodstream infection but also as a key pathway mediating nosocomial cross-transmission (9). Additionally, this strain exhibits distinct biological characteristics, including thermotolerance, biofilm formation, and cell aggregation (10, 11); among these, biofilm formation and aggregation can substantially enhance its tolerance to various antifungal agents (12). Currently, the core therapeutic agents for clinical fungal infections are predominantly traditional antifungal drugs, including azoles, polyenes, and echinocandins. *C. auris* represents the first fungal pathogen that may display pan-antifungal resistance to all clinically commonly utilized antifungal drugs (13–15). Meanwhile, these traditional antifungal agents generally suffer from inherent limitations, such as high toxicity, significant drug-drug interactions, high therapeutic costs, and limited bioavailability of certain agents, limitations that pose formidable challenges to clinical anti-infective therapy (16). Therefore, the development of novel antifungal drugs and the exploration of innovative therapeutic strategies hold profound clinical value and research significance.

Baicalein (BE), a naturally occurring flavonoid compound and the principal active component of *Scutellaria baicalensis* Georgi, possesses a polyphenolic structure and exhibits multiple pharmacological activities, including antioxidant, anti-inflammatory, antibacterial, and antifungal effects, with no apparent toxicity to normal somatic cells (17, 18). Unlike conventional azole antifungal agents such as fluconazole, BE is chemically distinct, and its antifungal mechanisms remain incompletely understood. Previous studies have demonstrated that BE can inhibit the proliferation of *Candida albicans* in a dose-dependent manner; when combined with quercetin, it can also reduce biofilm formation and hyphal growth of *C. albicans*, thereby displaying promising antifungal potential (19, 20). Preliminary studies conducted by our research group have confirmed that BE can effectively inhibit the proliferation of *C. auris* and exert potent antifungal activity (21, 22). However, because the pathogenicity and virulence of *C. auris* are regulated by multiple interconnected factors, the precise molecular mechanisms underlying the antifungal and anti-virulence effects of BE remain poorly understood and require further systematic investigation.

In this study, we systematically investigated the biological characteristics, adhesion behaviors, and regulatory mechanisms of *C. auris*. BE exhibited significant antifungal activity against both the yeast-type strain BJCA001 and four aggregated isolates. Moreover, BE suppressed the flocculation phenotype, reduced cell surface hydrophobicity (CSH), and downregulated the activities of key virulence factors, including proteases and phospholipases, in a concentration-dependent manner. BE also improved the survival of *Galleria mellonella* larvae, protected the host against fungal infection, and markedly inhibited the adhesion of *C. auris* to both biological and non-biological surfaces. In addition, BE not only inhibited biofilm formation but also exerted disruptive effects on mature biofilms. Transcriptomic analysis further revealed that BE regulated the expression of 11 adhesion- and biofilm-associated genes, including *SCF1* and *ALS1*, and that these transcriptional alterations were closely associated with impaired adhesion and biofilm formation capacities of *C. auris*.

In parallel, we systematically characterized the adhesion and biofilm colonization behaviors of *C. auris* on diverse biological and non-biological materials. Standardized *in vitro* adhesion models were established, and multiple analytical approaches were employed to quantitatively and qualitatively assess fungal adhesion on different material surfaces. Based on these findings, a standardized evaluation strategy for fungal adhesion was developed, which clarified the adhesion patterns of *C. auris* across different surface types. Collectively, these findings provide important experimental evidence for understanding the environmental adaptation and persistence mechanisms of this emerging pathogenic fungus.

## RESULTS

### BE significantly inhibited the growth of *C. auris*

**Fig 1:**
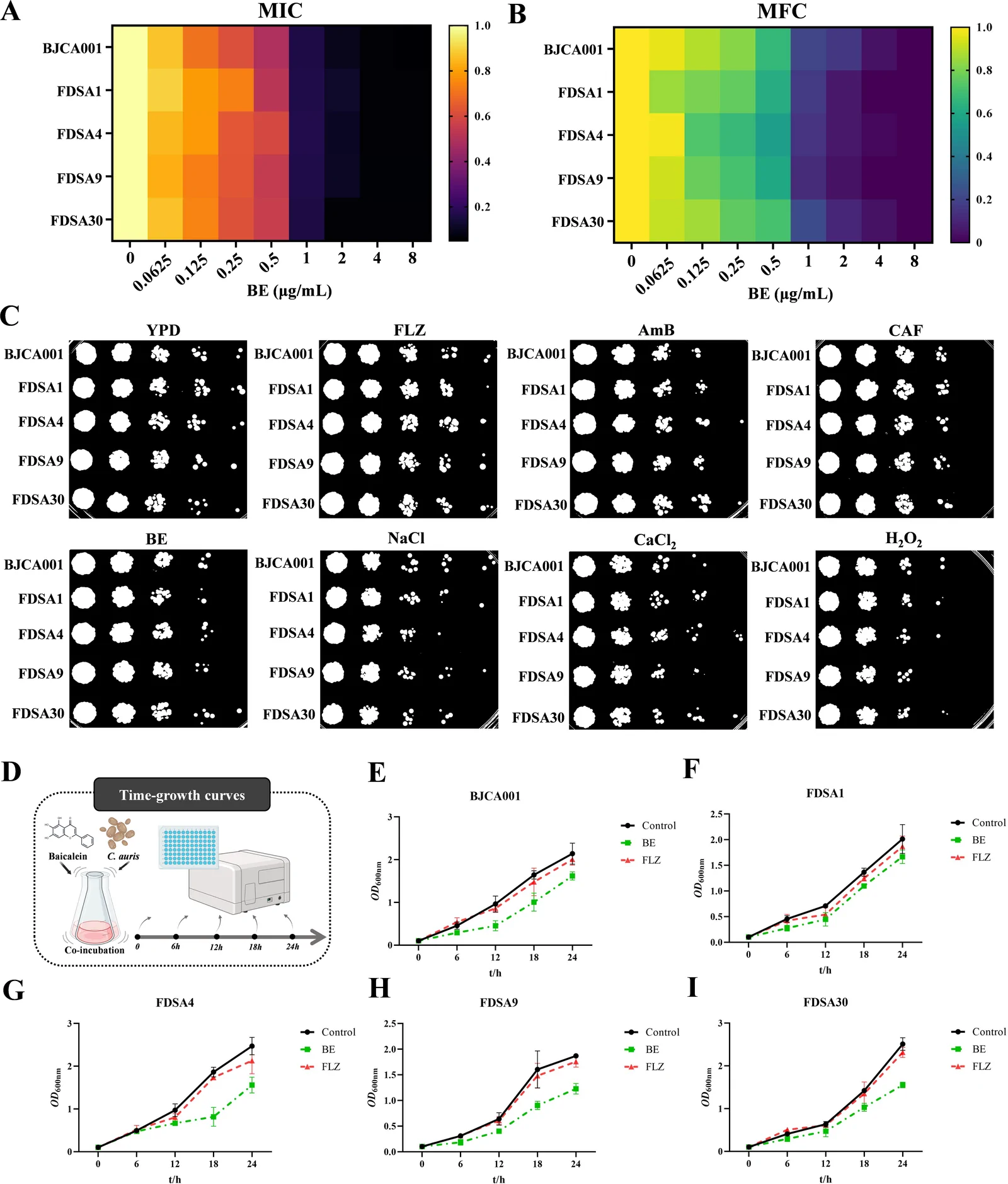
BE inhibited the growth of different *C. auris* isolates, including the single-celled and aggregated states. (**A**) Heatmap of MICs of BE against five *C. auris* strains. (**B**) Heatmap of MFCs of BE against five *C. auris* strains. (**C**) Spot assay. Five microliters of *C. auris* was spotted on YPD plates containing calcium chloride (700 mM), BE (8 μg/mL), sodium chloride (2 M), caspofungin (2 μg/mL), hydrogen peroxide (8 mM), fluconazole (32 μg/mL), and amphotericin B (2 μg/mL). Incubate at 30°C for 24 h and photograph for record. (**D–I**) Time growth curves in five *C. auris* strains. *C. auris* cultures were adjusted to OD_600_ = 0.1 and assigned to groups (control, BE-H, FLZ) as in Table 2. Cultures were incubated at 30°C with shaking (200 rpm), and OD_600_ was measured at 0, 6, 12, 18, and 24 h.

In the current study, the minimum inhibitory concentrations (MICs) and minimum fungicidal concentrations (MFCs) of BE against the yeast-type *C. auris* strain BJCA001 and four aggregative isolates (FDSA1, FDSA4, FDSA9, and FDSA30) were initially determined. The results revealed that the MICs for all tested strains were consistently 1 μg/mL; specifically, the MFCs of BJCA001, FDSA4, and FDSA30 were 8 μg/mL, whereas the MFCs of FDSA1 and FDSA9 were 4 μg/mL (Fig. 1A and B). In addition, all five strains had a sessile minimum inhibitory concentration (SMIC) of 32 μg/mL (Table 1). Collectively, these observations illustrated that BE exerted significant inhibitory activity against all five *C. auris* strains, thereby laying an important foundation for the subsequent experiments.

**TABLE 1:** MIC, MFC, and SMIC of BE against *C. auris^a^*

Strain	Reference or source	MIC (μg/mL)	MFC (μg/mL)	SMIC (μg/mL)
BJCA001 (Clade I)	Bing, Jian et al., *Nat Commun* (11)	1	8	32
FDSA1 (Clade I)	Bing, Jian et al., *Nat Commun* (11)	1	4	32
FDSA4 (Clade I)	Bing, Jian et al., *Nat Commun* (11)	1	8	32
FDSA9 (Clade I)	Bing, Jian et al., *Nat Commun* (11)	1	4	32
FDSA30 (Clade I)	Bing, Jian et al., *Nat Commun* (11)	1	8	32

^*a*^ MIC, minimum inhibitory concentration; MFC, minimum fungicidal concentration; SMIC, sessile minimum inhibitory concentration.

To examine the growth-inhibitory effects of BE on the yeast-type *C. auris* strain BJCA001 and four aggregative isolates (FDSA1, FDSA4, FDSA9, and FDSA30), we constructed a panel of stress-sensitive media. This experimental setup was designed to compare the phenotypic responses induced by BE with those triggered by conventional antifungal agents and representative environmental stress conditions. This series of media was supplemented with calcium chloride (CaCl_2_) (700 mM), BE (8 μg/mL), sodium chloride (NaCl) (2 M), caspofungin (CAF) (2 μg/mL), hydrogen peroxide (H_2_O_2_) (8 mM), fluconazole (FLZ) (32 μg/mL), and amphotericin B (AMB) (2 μg/mL). The selected stressors encompassed classic antifungal agents and various non-specific stress factors, with the purpose of systematically assessing the antifungal activity of BE against the aforementioned *C. auris* strains via multi-dimensional comparative analysis. The results showed that compared with the YPD medium without stressors, there was no significant difference in the growth of *C. auris* in the FLZ medium. The growth of BJCA001, FDSA1, and FDSA9 was inhibited in the AMB medium. The growth of all *C. auris* strains was inhibited in the CAF medium. In the BE medium, the growth of all strains was inhibited except FDSA30. The growth of FDSA4 and FDSA30 was inhibited in the NaCl medium. The growth of BJCA001, FDSA1, and FDSA9 was inhibited in the CaCl_2_ medium. The growth of all strains was inhibited in the H_2_O_2_ medium, among which FDSA9 showed the most significant inhibition (Fig. 1C). These results suggested that, except for FLZ, the other stressors exerted certain inhibitory effects on *C. auris*. Among them, CAF and H_2_O_2_ exhibited the most significant inhibitory effects, while BE also displayed antifungal activity comparable to these two stressors.

Ultimately, time-growth curves of the five *C. auris* strains treated with BE were established in the current study to assess the sustainability of its inhibitory activity against *C. auris*. Data from the time-kill curves revealed that BE exerted a weak inhibitory effect on *C. auris* growth during the early cultivation phase (0–6 h); the inhibitory effect was most pronounced between 12 and 18 h of cultivation. Following 18 h of cultivation, the inhibitory effect gradually diminished; however, the overall inhibitory efficacy remained significantly superior to that observed in both the control group and the FLZ group (Fig. 1D through I).

Based on the findings from the aforementioned experiments, the yeast-type strain BJCA001 and the aggregative strain FDSA9 were selected for subsequent experiments, and the experimental groups were established according to Table 2.

**TABLE 2:** Experimental groups and intervention methods

Experimental groups	Intervention methods
Group A: control	*C. auris* (BJCA001 or FDSA9) + RPMI-1640
Group B: (8 μg/mL) BE-L	*C. auris* (BJCA001 or FDSA9) + 16 µg/mL BE (fungal suspension: drug solution = 1:1)
Group C: (16 μg/mL) BE-M	*C. auris* (BJCA001 or FDSA9) + 32 µg/mL BE (fungal suspension: drug solution = 1:1)
Group D: (32 μg/mL) BE-H	*C. auris* (BJCA001 or FDSA9) + 64 µg/mL BE (fungal suspension: drug solution = 1:1)
Group E: (32 μg/mL) FLZ	*C. auris* (BJCA001 or FDSA9) + 64 µg/mL FLZ (fungal suspension: drug solution = 1:1)

### BE inhibited multiple virulence factors of *C. auris* and safeguards the host against its invasion

**Fig 2:**
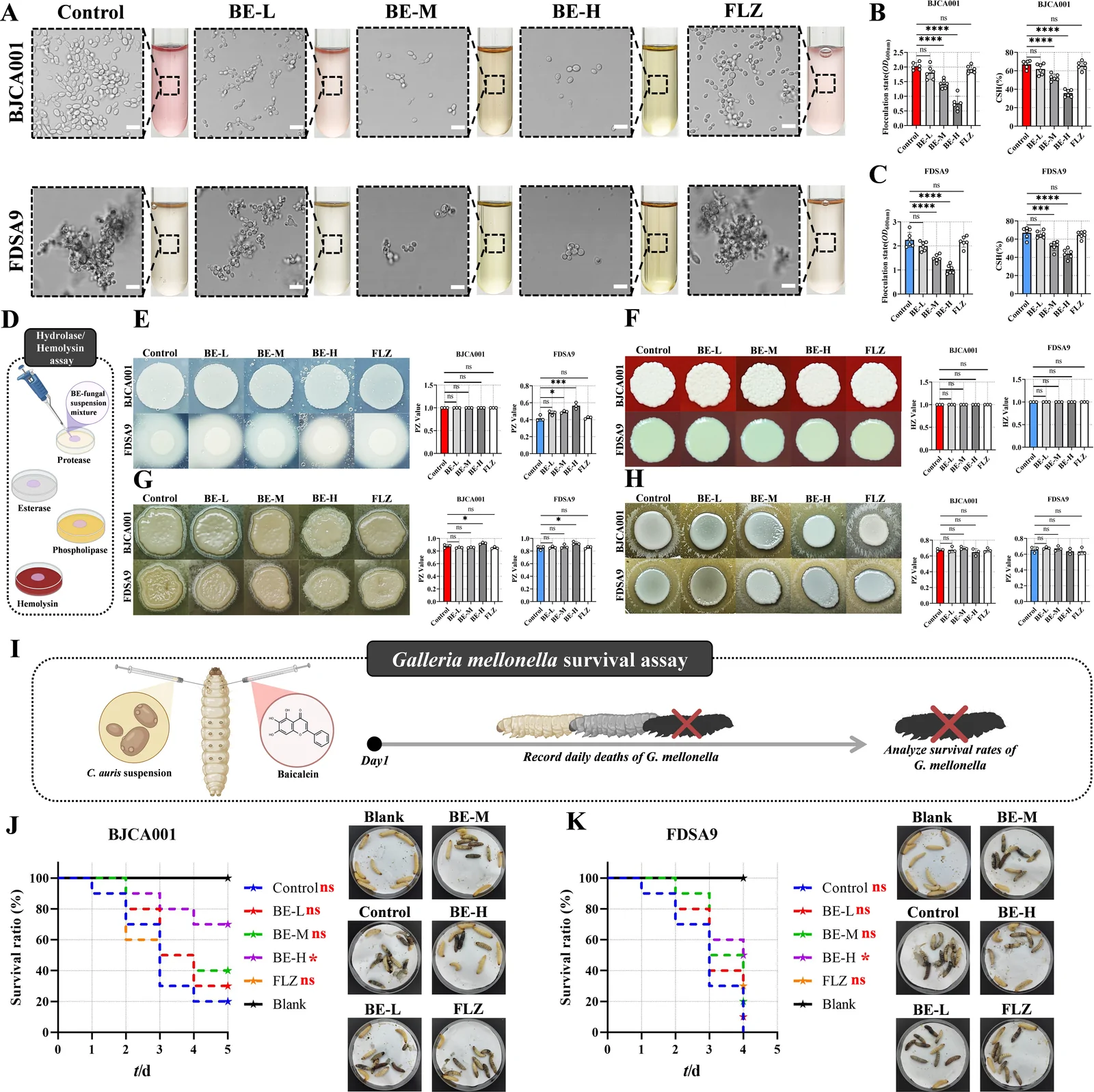
BE suppressed virulence factor expression in *C. auris* and confers host protection against fungal invasion. (**A**) Flocculation experiment and microscopic images. *C. auris* was adjusted to 2 × 10^6^ CFU/mL. After 24 h of incubation at 30°C, flocculation was photographed and OD_600_ measured. Fungal suspensions were then loaded onto confocal dishes and imaged using a DMi8 microscope. Scale bars: 10 μm. (**B**) The OD_600_ of BJCA001 in the flocculation state and the CSH of BJCA001 were determined as follows. *C. auris* suspensions were adjusted to OD_600_ = 0.4 (A0). Subsequently, 1.2 mL of each fungal suspension was transferred to an EP tube, followed by the addition of 200 μL of hexadecane. The mixtures were incubated at room temperature for 10 min and then vortexed at maximum speed for 1 min. After standing for an additional 15 min to allow phase separation, the OD_600_ of the lower aqueous phase (A1) was measured. CSH was calculated using the following formula: CSH = [1 − (A1 / A0)] × 100%. (**C**) The OD_600_ of FDSA9 in the flocculation state; the CSH of FDSA9, see panel B for details. (**D**) Hydrolase and hemolysin experiment. Ten microliters of the mixed solution was spotted onto the culture medium and incubated at 30°C for 3–4 days. Hydrolase activity was assessed by the precipitation halo and colony diameter: colony diameter / (precipitation halo + colony diameter). (**E**) Protease activity and quantification of BJCA001 and FDSA9. (**F**) Hemolysin activity and quantification of BJCA001 and FDSA9. (**G**) Phospholipase activity and quantification of BJCA001 and FDSA9. (**H**) Esterase activity and quantification of BJCA001 and FDSA9. (**I**) *G. mellonella* survival experiment. For each group, 10 larvae were randomly selected and injected in the right posterior paraventral foot with 10 μL of *C. auris* (7.5 × 10^6^ CFU/mL). After 2 h, 10 μL of PBS, BE (8, 16, or 32 μg/mL), or FLZ (32 μg/mL) was injected into the left posterior paraventral foot. Larvae were then incubated in the dark at 37°C for 5 days, with daily mortality recorded. (**J**) Survival experiment of *G. mellonella* infected with BJCA001. (**K**) Survival experiment of *G. mellonella* infected with FDSA9. All groupings are shown in Table 2. All data are presented as mean ± SD: ns, not significant; *P < 0.05; ***P < 0.001; ****P < 0.0001 vs the control group.

After confirming the significant growth-inhibitory activity of BE against *C. auris*, we explored whether BE exerted inhibitory effects on the virulence factors of *C. auris*. Initially, we explored the impact of BE on the flocculation phenotype of *C. auris*. Data obtained from *in vitro* EP tube assays demonstrated that as the concentration of BE increased, the flocculation capacity of *C. auris* was significantly suppressed, with the solution clarity of the fungal suspension increasing in a concentration-dependent manner (Fig. 2A). Microscopic observations confirmed that elevated BE concentrations effectively inhibited the proliferation and aggregation capacities of *C. auris* (Fig. 2A). These experimental findings were highly consistent with the OD_600_ absorbance measurements (Fig. 2B and C), thereby validating the inhibitory effect of BE on the flocculation state of *C. auris*. BE-M and BE-H groups showed the strongest effects; no significant inhibition was observed in the FLZ group. Subsequently, alterations in the CSH of *C. auris* were evaluated in the current study. Experimental data demonstrated that the CSH of *C. auris* was significantly reduced in a BE concentration-dependent manner. Among them, BE-M and BE-H groups showed the strongest effects, whereas the FLZ group did not (Fig. 2B and C).

Furthermore, we explored the effects of BE on the activities of three hydrolases and hemolysin (Fig. 2D). The results of protease activity assays showed that strain BJCA001 exhibited no protease activity, whereas strain FDSA9 displayed extremely strong protease activity. Moreover, the protease activity of FDSA9 decreased significantly as the BE concentration increased, with the inhibitory effects being statistically significant in the BE-M and BE-H groups but not in the FLZ group (Fig. 2E). Results of hemolysin activity assays indicated that neither of the two strains exhibited hemolysin activity (Fig. 2F). For phospholipase activity detection, both strains BJCA001 and FDSA9 showed strong phospholipase activity, and the phospholipase activity of both strains was significantly reduced with the elevation of BE concentration, with the inhibitory effect in the BE-H group being statistically significant. However, the FLZ group did not show a significant inhibitory effect (Fig. 2G). The results of esterase activity assays demonstrated that both strains possessed strong esterase activity, but BE had no significant effect on their esterase activity (Fig. 2H).

Ultimately, we utilized a *G. mellonella* infection model to explore whether BE could enhance the survival rate of *G. mellonella* via the exertion of a host-protective effect (Fig. 2I). Experimental data revealed that the survival rate of *G. mellonella* in the groups infected with both strains exhibited an upward trend with escalating BE concentrations; however, this survival-promoting effect was only statistically significant in the BE-H group; no significant effect was observed in the FLZ group (Fig. 2J and K).

### BE inhibited *C. auris* adhesion to biological surfaces

Following the confirmation of BE-mediated inhibition of diverse *C. auris* virulence factors, we focused on adhesion, the most conserved and critical virulence determinant of this pathogen.

**Fig 3:**
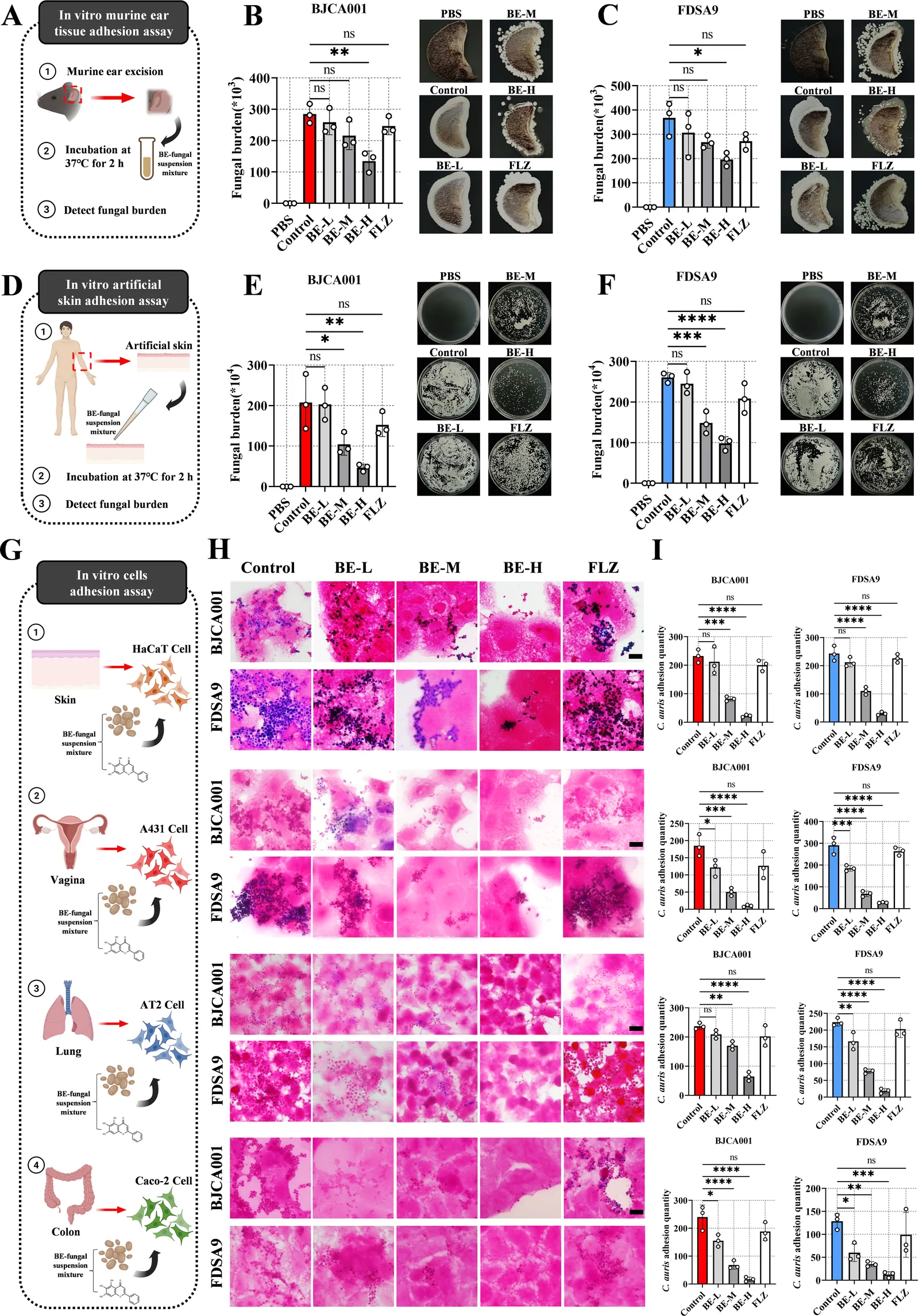
BE inhibited the adhesion of *C. auris* to murine skin, artificial skin, and various human epithelial cell types. (**A**) *In vitro* murine ear tissue adhesion assay. Ears of C57BL/6 mice were trimmed and washed with PBS. *C. auris* suspension was adjusted to 2 × 10^6^ CFU/mL. Each ear was incubated with the suspension at 37°C, 80 rpm, for 2 h. After incubation, ears were washed one to two times with PBS to remove non-adherent cells, then either plated on YPD for imaging or processed for CFU quantification. (**B**) Adhesion of BJCA001 to murine ears and quantification. (**C**) Adhesion of FDSA9 to murine ears and quantification. (**D**) *In vitro* artificial skin adhesion assay. *C. auris* was adjusted to 2 × 10^6^ CFU/mL. A total 1.5 mL of the suspension was applied to artificial skin and evenly spread with a sterile cotton swab, followed by 2 h incubation at 37°C. After incubation, non-adherent cells were removed by one to two PBS washes, and fungal load was quantified via plate coating assay. (**E**) Adhesion of BJCA001 to artificial skin and quantification. (**F**) Adhesion of FDSA9 to artificial skin and quantification. (**G**) *In vitro* cell adhesion assay. In each well of a six-well plate, 2 mL of HaCaT (or A431, AT2, or Caco-2) cells (5 × 10^5^ cells/mL) were seeded and cultured at 37°C and 5% CO_2_ for 24 h. *C. auris* suspensions were then adjusted to 2 × 10^6^ CFU/mL. Plates were incubated at 37°C and 5% CO_2_ for 4 h. Cells were washed one to two times with PBS, fixed with 4% paraformaldehyde at room temperature for 20 min, washed again with PBS, and stained using an enhanced Gram staining kit. *C. auris* adhesion was visualized under an ECLIPSE Ni microscope. (**H and I**) The adhesion of BJCA001 and FDSA9 to different human epithelial cells and quantification. Scale bars: 20 μm. All groupings are shown in Table 2. All data are presented as mean ± SD: ns, not significant; *P < 0.05; **P < 0.01; ***P < 0.001; ****P < 0.0001 vs the control group.

First, adhesion assays were performed at the organismal level using a murine ear skin model. The results demonstrated that the number of adherent cells of both strains on the murine auricular surface decreased progressively with increasing BE concentrations. This reduction reached statistical significance exclusively in the BE-H group, whereas no significant difference was observed in the FLZ group (Fig. 3A through C).

Subsequently, an artificial skin adhesion assay was conducted. A concentration-dependent decrease in the adherent cell counts of both strains was observed on the artificial skin surface. This inhibitory effect was statistically significant in both the BE-M and BE-H groups, in contrast to the FLZ group, which exhibited no significant activity (Fig. 3D through F).

Finally, adhesion experiments of *C. auris* to human epithelial cells were carried out (Fig. 3G): results from the HaCaT cell adhesion assay revealed that the number of both *C. auris* strains adhering to the surface of HaCaT cells gradually decreased with increasing BE concentrations, with statistically significant inhibitory effects observed in the BE-M and BE-H groups and no significant difference in the FLZ group (Fig. 3H and I). Results from the A431 cell adhesion assay demonstrated that elevated BE concentrations significantly reduced the number of both *C. auris* strains adhering to the surface of A431 cells, and all BE-treated groups exhibited statistical significance, while the FLZ group showed no significance (Fig. 3H and I). Results from the AT2 cell adhesion assay indicated that the number of both *C. auris* strains adhering to the surface of AT2 cells gradually decreased with increasing BE concentrations; for strain BJCA001, the inhibitory effect was statistically significant in the BE-M and BE-H groups, whereas for strain FDSA9, the inhibitory effect was statistically significant in all BE-treated groups, and the FLZ group showed no significant effect in either strain (Fig. 3H and I). Results from the Caco-2 cell adhesion assay demonstrated that elevated BE concentrations significantly reduced the number of both *C. auris* strains adhering to the surface of Caco-2 cells, with all BE-treated groups showing statistical significance and no significant difference observed in the FLZ group (Fig. 3H and I).

### BE inhibited *C. auris* adhesion to non-biological material surfaces

**Fig 4:**
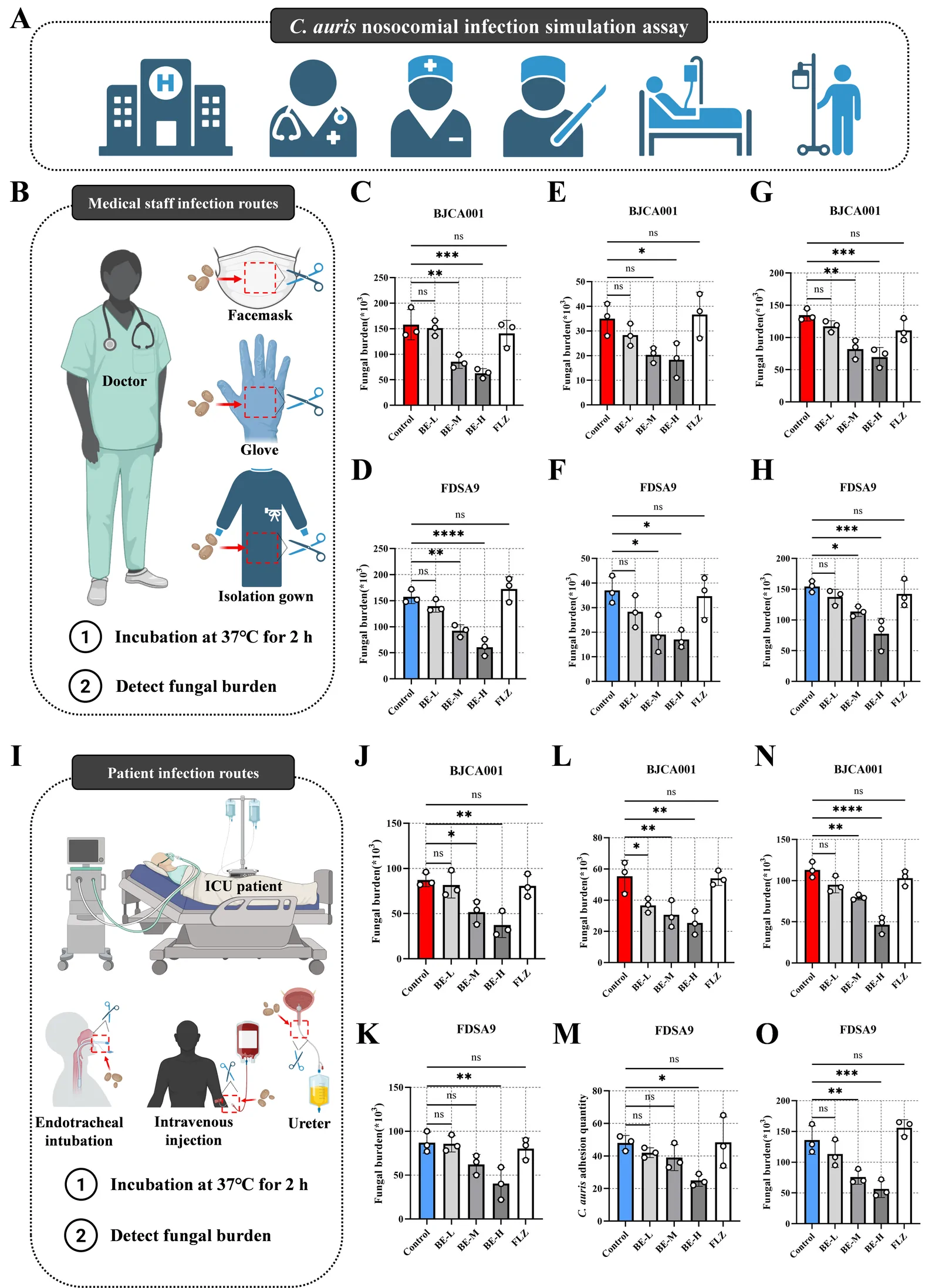
BE inhibited the adhesion of *C. auris* to medical items (non-biological materials). (**A**) This experiment simulated the adhesion of *C. auris* in a medical environment. (**B**) *C. auris* concentration was adjusted to 2 × 10^6^ CFU/mL. A total of 1.5 mL of the suspension was applied to each medical supply—mask (PP), isolation gown (polypropylene non-woven fabric), and gloves (nitrile)—and evenly spread with a sterile cotton swab. Samples were incubated at 30°C for 2 h, then washed one to two times with PBS to remove unattached cells. *C. auris* was recovered from the surfaces for plate counting to quantify fungal load. (**C and D**) BJCA001 and FDSA9 adhesion to masks. (**E and F**) BJCA001 and FDSA9 adhesion to gloves. (**G and H**) BJCA001 and FDSA9 adhesion to isolation gowns. (**I**) Same method as panel B, using infusion tube (PVC), endotracheal tube (silicone), and urinary catheter (latex) as adhering materials. (**J and K**) BJCA001 and FDSA9 adhesion to infusion tubes. (**L and M**) BJCA001 and FDSA9 adhesion to endotracheal tubes. (**N and O**) BJCA001 and FDSA9 adhesion to isolation urinary catheters. All groupings are shown in Table 2. All data are presented as mean ± SD: ns, not significant; *P < 0.05; **P < 0.01; ***P < 0.001; ****P < 0.0001 vs the control group.

After confirming the inhibitory effect of BE on the adhesion of *C. auris* to biological organisms, we investigated whether BE could abrogate the adhesion of *C. auris* to non-biological materials. To simulate clinical nosocomial infection settings, six categories of medical devices frequently utilized by healthcare providers and ICU patients were selected for adhesion assays (Fig. 4A, B and I), and the experimental findings were summarized as follows: BE significantly suppressed the adhesion of both *C. auris* strains to mask surfaces, with the inhibitory effect achieving statistical significance in both the BE-M and BE-H groups (Fig. 4C and D); BE exerted an inhibitory effect on the adhesion of both *C. auris* strains to disposable glove surfaces. Specifically, strain BJCA001 exhibited a statistically significant reduction in adhesion only in the BE-H group, whereas strain FDSA9 displayed statistically significant inhibitory effects in both the BE-M and BE-H groups (Fig. 4E and F); BE significantly inhibited the adhesion of both *C. auris* strains to isolation gown surfaces, with the inhibitory effects in the BE-M and BE-H groups being statistically significant (Fig. 4G and H).

Additionally, BE suppressed the adhesion of both *C. auris* strains to endotracheal tube surfaces: strain BJCA001 showed statistically significant inhibitory effects in both the BE-M and BE-H groups, while strain FDSA9 exhibited a statistically significant inhibitory effect exclusively in the BE-H group (Fig. 4J and K); BE exerted an inhibitory effect on the adhesion of both *C. auris* strains to infusion tube surfaces. For strain BJCA001, the inhibitory effect was statistically significant across all BE-treated groups, whereas strain FDSA9 displayed a statistically significant inhibitory effect only in the BE-H group (Fig. 4L and M). Furthermore, BE significantly suppressed the adhesion of both *C. auris* strains to ureteral catheter surfaces, with the inhibitory effects in the BE-M and BE-H groups achieving statistical significance (Fig. 4N and O). Notably, FLZ failed to exert any inhibitory effect on the adhesion of *C. auris* to the surfaces of all aforementioned non-biological materials.

### BE inhibited *C. auris* biofilms

Subsequent to confirming that BE effectively inhibited the adhesive capacity of *C. auris* to both biological organisms and non-biological materials, we expanded the research scope, enhanced the research depth, and focused on biofilms—a key virulence structure formed following adhesion.

**Fig 5:**
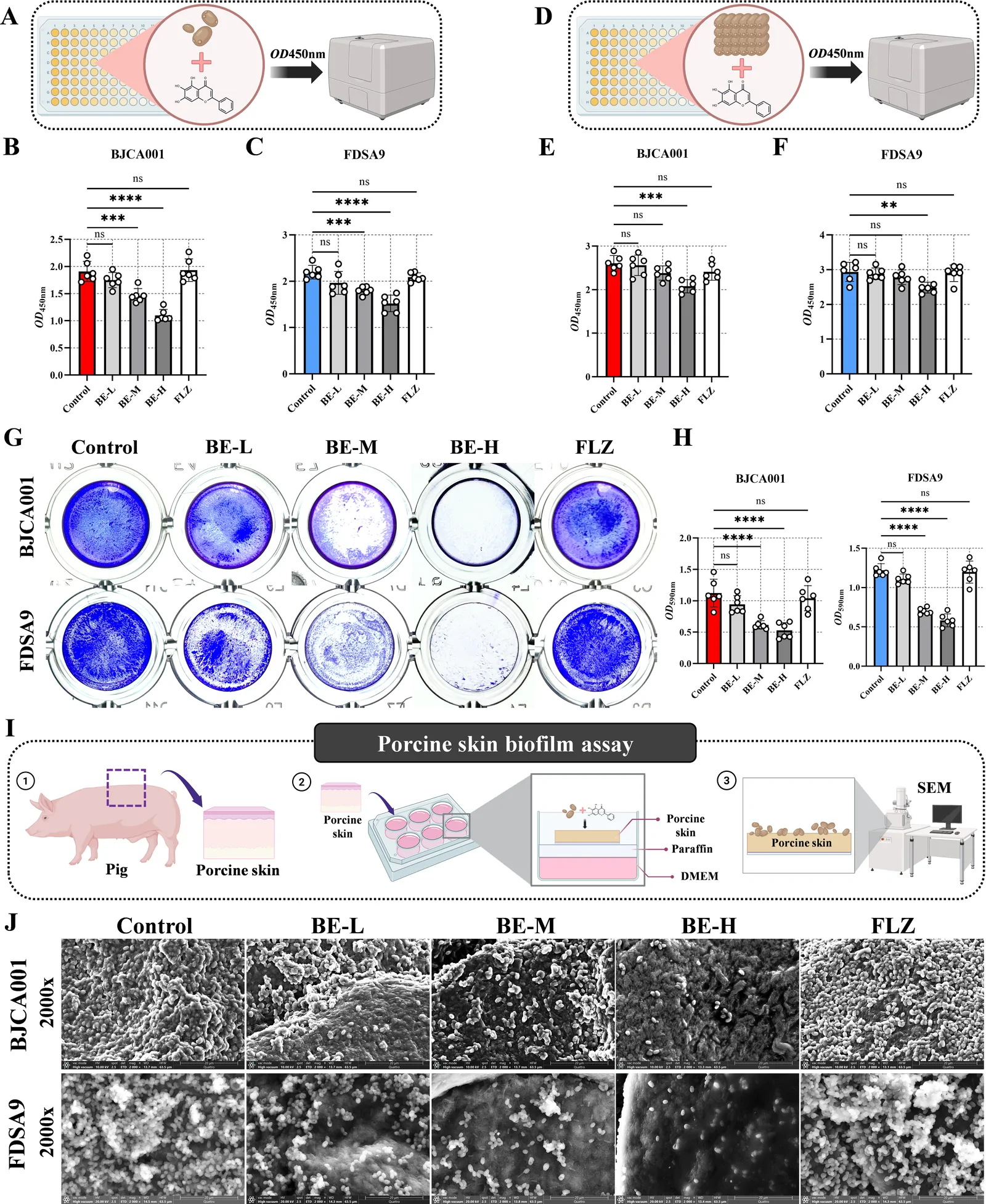
BE inhibited the biofilms of *C. auris*. (**A–C**) BE-mediated inhibition and quantification of metabolic activity during *C. auris* biofilm formation. The fungal suspension was adjusted to 2 × 10^6^ CFU/mL. Each well received 100 μL of fungal suspension and 100 μL of drug solution, followed by 24 h incubation at 30°C. After incubation, supernatants were removed and planktonic cells washed with PBS. Then, 50 μL of XTT/menadione solution was added per well and incubated in the dark for 2 h. Absorbance was measured at 450 nm using a microplate reader. (**D–F**) BE inhibition and quantification of metabolic activity in mature *C. auris* biofilms. The fungal suspension was adjusted to 2 × 10^6^ CFU/mL. Each well received 100 μL of fungal suspension and was incubated at 30°C for 24 h to form biofilms. Then, 100 μL of drug solution was added, and incubation continued at 30°C for another 24 h. After incubation, the supernatant was removed and adherent cells were washed with PBS. Next, 50 μL of XTT/menadione solution was added to each well and incubated in the dark for 2 h. Absorbance was measured at 450 nm using a microplate reader. (**G and H**) Biofilm crystal violet assay and quantification. *C. auris* was adjusted to 2 × 10^6^ CFU/mL. After 24 h incubation at 30°C, samples were rinsed once with PBS, stained with 0.5% crystal violet for 30 min, rinsed two to three times with PBS, and *OD*_590_ measured using a microplate reader. (**I**) Porcine skin biofilm assay. Porcine skin was washed repeatedly with PBS and 75% ethanol, then depilated. Full-thickness (12 mm) skin samples were collected using a skin punch and placed in 12-well plates containing 3 mL DMEM. After 6 h incubation at 37°C and 5% CO_2_, samples were rinsed with PBS, air-dried, and transferred to fresh DMEM (1 mL paraffin barrier added at the medium–skin interface). *C. auris* suspensions were adjusted to 2 × 10^6^ CFU/mL; 20 μL was evenly applied to each skin surface and incubated for 24 h at 37°C, 5% CO_2_, and low humidity. Post-incubation, samples were fixed in ice-cold 2.5% glutaraldehyde for ≥2 h, dehydrated sequentially in 30%, 70%, 95%, and 100% ethanol (10 min each), air-dried, gold-coated, and imaged by SEM. (**J**) BE effect on *C. auris* biofilm formation on porcine skin (SEM). All groupings are shown in Table 2. All data are presented as mean ± SD: ns, not significant; **P < 0.01; ***P < 0.001; ****P < 0.0001 vs the control group.

Initially, the XTT assay was employed to assess the inhibitory effect of BE on the biofilm formation process of *C. auris* (Fig. 5A). Experimental results demonstrated that BE significantly suppressed biofilm formation in both *C. auris* strains, with the inhibitory effect achieving statistical significance in both the BE-M and BE-H groups, whereas no significant difference was detected in the FLZ group (Fig. 5B and C). Additionally, the destructive effect of BE on mature biofilms was evaluated (Fig. 5D); results indicated that BE exerted a modest destructive effect on the mature biofilms of both *C. auris* strains, though this effect was only statistically significant in the BE-H group, with no significant efficacy observed in the FLZ group (Fig. 5E and F).

Furthermore, crystal violet staining was utilized to quantify the biofilm biomass of *C. auris*. The findings revealed that BE effectively inhibited biofilm biomass production in both *C. auris* strains, with the inhibitory effect attaining statistical significance in both the BE-M and BE-H groups. In contrast, the FLZ group failed to exhibit a significant inhibitory effect on biofilm biomass (Fig. 5G and H).

Finally, a porcine skin biofilm model was established to assess *C. auris* biofilm formation on biological surfaces (Fig. 5I). Results demonstrated that BE effectively inhibited biofilm formation of both *C. auris* strains on porcine skin surfaces, and with increasing BE concentrations, the biofilms formed by both strains exhibited a progressive destruction trend (Fig. 5J).

### Transcriptomic analysis revealed the molecular mechanism of BE-mediated inhibition of *C. auris*

**Fig 6:**
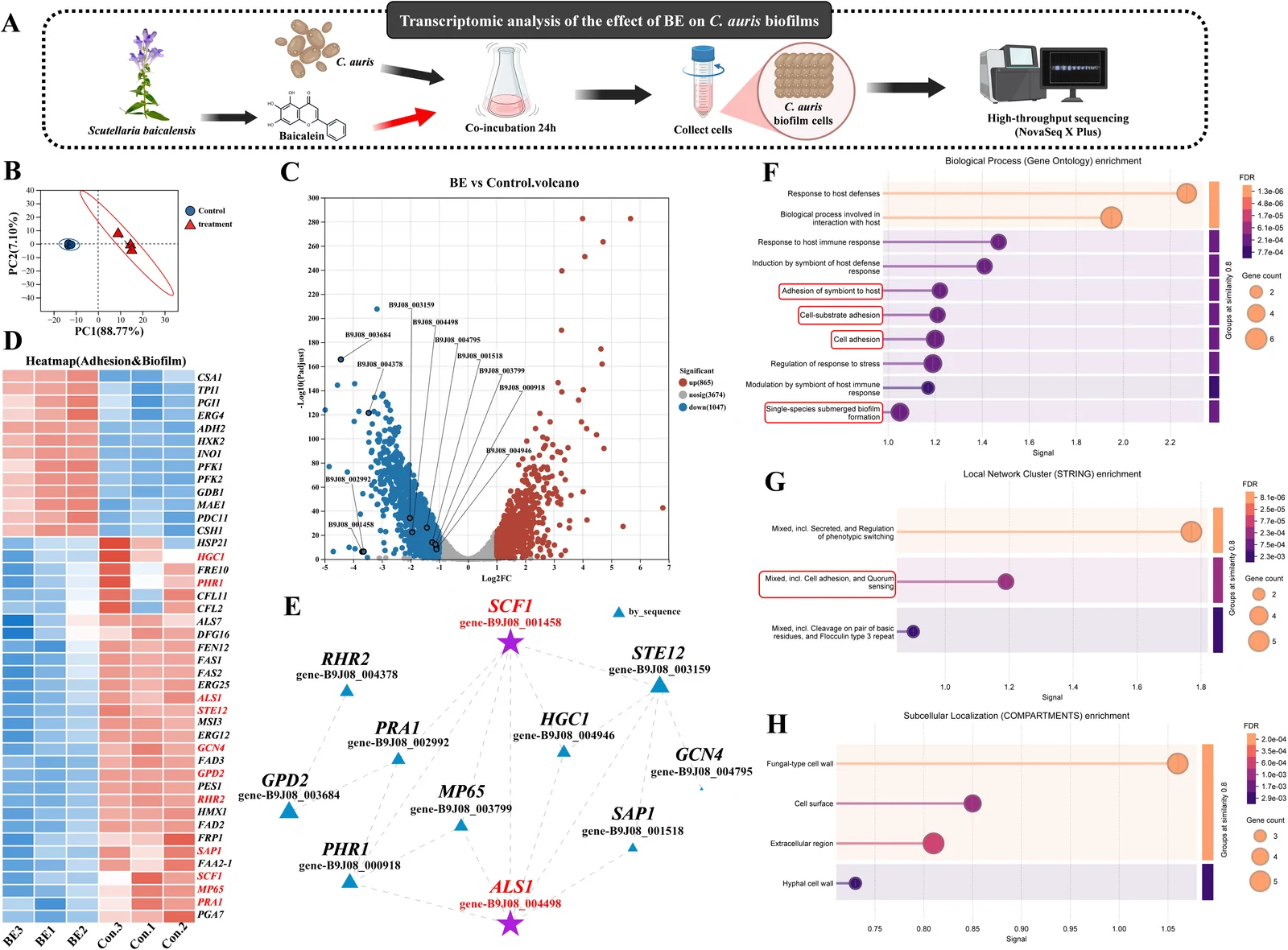
Transcriptomic analysis revealed the mechanism underlying BE-mediated inhibition of *C. auris*. (**A**) Total RNA was extracted from *C. auris* in control (*n* = 3) and BE (*n* = 3) groups for transcriptomic sequencing. (**B**) PCA analysis of samples. (**C**) Volcano plot. (**D**) Heatmap of adhesion and biofilm genes. (**E**) Protein interaction network (*PHR1*, B9J08_000918; *SCF1*, B9J08_001458; *SAP1*, B9J08_001518; *PRA1*, B9J08_002992; *STE12*, B9J08_003159; *GPD2*, B9J08_003684; *MP65*, B9J08_003799; *RHR2*, B9J08_004378; *ALS1*, B9J08_004498; *GCN4*, B9J08_004795; *HGC1*, B9J08_004946). (**F**) Biological process (Gene Ontology) enrichment. (**G**) Local network cluster (STRING) enrichment. (**H**) Subcellular localization (COMPARTMENTS) enrichment.

Synthesizing all the aforementioned results, we have confirmed that BE exerted a significant inhibitory effect on *C. auris*, with its inhibitory effects encompassing multiple virulence factors such as adhesion and biofilms. To explore the molecular mechanism underlying the inhibitory effect of BE on *C. auris*, transcriptomic sequencing analysis was performed in this study (Fig. 6A).

Results from the volcano plot and principal component analysis (PCA) demonstrated significant differences in the transcriptomes between BE-treated and untreated *C. auris* groups (Fig. 6B and C). To delve deeper into the underlying molecular mechanism, we focused on all genes associated with adhesion and biofilm formation. Heatmap analysis revealed that these genes exhibited significant changes in expression following BE treatment (Fig. 6D).

After screening and optimization of the aforementioned adhesion- and biofilm-related genes, 11 target genes (*PHR1*, B9J08_000918; *SCF1*, B9J08_001458; *SAP1*, B9J08_001518; *PRA1*, B9J08_002992; *STE12*, B9J08_003159; *GPD2*, B9J08_003684; *MP65*, B9J08_003799; *RHR2*, B9J08_004378; *ALS1*, B9J08_004498; *GCN4*, B9J08_004795; *HGC1*, B9J08_004946) were ultimately selected for protein-protein interaction network analysis (Fig. 6E). The results indicated that the *SCF1* and *ALS1* genes play a dominant regulatory role.

Subsequently, Gene Ontology enrichment analysis, Local Network Cluster (STRING) analysis, and Subcellular Localization (COMPARTMENTS) enrichment analysis were performed on these 11 target genes (Fig. 6F through H). The results showed that the changes in their expression were associated with “Adhesion of symbiont to host,” “Cell-substrate adhesion,” “Cell adhesion,” “Single-species submerged biofilm,” and “Mixed, incl. cell adhesion, and quorum sensing.”

## DISCUSSION

*Candida auris* is an emerging multidrug-resistant fungal pathogen characterized by strong environmental persistence, efficient skin colonization, and rapid nosocomial transmission. Adhesion and biofilm formation are considered critical virulence traits that enable long-term colonization on both host tissues and medical-device surfaces, thereby contributing to healthcare-associated infections and outbreak dissemination (23–25). In addition, increasing evidence suggests that aggregated phenotypes of *C. auris* exhibit enhanced adhesion capacity, biofilm formation, and virulence compared with yeast-type isolates, highlighting the importance of targeting adhesion-associated processes as a potential antifungal strategy (11).

In the present study, BE exhibited potent antifungal activity against both yeast-type and aggregated *C. auris* isolates. The consistent MIC and SMIC values observed across different phenotypic strains suggest that the antifungal efficacy of BE is relatively independent of strain heterogeneity. Moreover, BE maintained inhibitory activity under multiple stress conditions and displayed sustained time-dependent antifungal effects, supporting its potential as a broad-spectrum anti-*C*. *auris* agent.

Beyond growth inhibition, BE markedly attenuated several virulence-associated phenotypes of *C. auris*, including flocculation, CSH, and hydrolytic enzyme activities. Flocculation and elevated CSH are closely associated with fungal aggregation, adhesion, and biofilm development (26–29), while hydrolytic enzymes facilitate host invasion and tissue colonization (30–32). Our findings demonstrated that BE significantly reduced flocculation and CSH in a concentration-dependent manner and selectively inhibited protease and phospholipase activities. These results suggest that BE may impair fungal pathogenicity by modulating cell surface-associated virulence properties. Furthermore, the improved survival of *G. mellonella* following BE treatment indicates that the inhibitory effects of BE on fungal virulence may translate into host-protective activity *in vivo*.

Adhesion is the initial and indispensable step in *C. auris* colonization and biofilm establishment (33, 34). In this study, BE broadly inhibited the adhesion of *C. auris* to multiple biological and non-biological substrates, including epithelial cell lines, skin models, and clinically relevant medical-device materials. Importantly, the anti-adhesive effects of BE were consistently observed across diverse surface environments, suggesting that its activity is not restricted to a specific substrate type. Since surface colonization is a major prerequisite for persistent environmental contamination and nosocomial transmission, the ability of BE to interfere with adhesion may represent an important advantage for infection prevention and control.

Biofilm formation is another critical virulence characteristic of *C. auris* that contributes to increased tolerance to antifungal agents, enhanced resistance to host immune clearance, and improved environmental persistence (35). We demonstrated that BE significantly inhibited biofilm formation and reduced biofilm biomass in multiple experimental models. Although BE exerted only partial disruptive effects on mature biofilms, this observation is consistent with the highly compact and drug-tolerant architecture of established fungal biofilms, which often limits antimicrobial penetration. Nevertheless, the ability of BE to interfere with early-stage biofilm development may still have important clinical implications, as prevention of biofilm establishment is generally more achievable than eradication of mature biofilms.

Transcriptomic analysis further revealed that the antifungal and anti-virulence effects of BE were closely associated with alterations in adhesion- and biofilm-related gene expression. Among the identified target genes, *SCF1* and *ALS1* appeared to occupy central positions within the regulatory network. *SCF1* has recently been identified as a *C. auris*-specific adhesin involved in colonization of both hydrophobic and hydrophilic surfaces (28), whereas *ALS1* contributes to fungal adhesion and biofilm maturation (36). Previous studies have further demonstrated that *SCF1* is closely associated with aggregate formation in *C. auris*, as aggregated isolates exhibit significant upregulation of adhesion-related genes involved in cell-cell adhesion and multicellular biofilm development. In particular, *SCF1* has been recognized as a key regulator mediating intercellular adhesion, aggregate formation, and surface colonization (7). Since aggregated phenotypes are believed to enhance environmental persistence, stress tolerance, and virulence of *C. auris*, the significant downregulation of *SCF1* following BE treatment suggests that BE may interfere not only with initial surface attachment but also with multicellular aggregation-associated adaptive processes. Therefore, disruption of *SCF1*-mediated adhesion networks may represent one of the core molecular mechanisms underlying the anti-*C*. *auris* activity of BE.

The anti-adhesive activity of BE may also be linked to its effects on cell surface properties. We observed that BE significantly reduced cell surface hydrophobicity and suppressed the flocculation phenotype, both of which are closely related to fungal adhesion and intercellular aggregation. Therefore, it is plausible that BE disrupts the initial attachment process of *C. auris* by altering cell wall-associated adhesive characteristics and interfering with adhesion-related transcriptional networks. In turn, impaired cell-cell interactions and reduced aggregate formation may indirectly inhibit biofilm development and environmental persistence. These findings collectively suggest that BE exerts its anti-virulence effects through coordinated regulation of adhesion, aggregation, and biofilm-associated pathways.

Several limitations should be acknowledged in this study. Although transcriptomic analysis identified multiple candidate target genes associated with adhesion and biofilm regulation, direct functional validation was not performed. In addition, the precise active components of BE and their molecular targets remain unclear. Furthermore, the present work primarily relied on *in vitro* models and the *G. mellonella* infection system, and further validation in mammalian infection models will be necessary to evaluate the therapeutic efficacy and biosafety of BE *in vivo*. Future studies should therefore focus on identifying the active antifungal constituents of BE and clarifying their direct interactions with adhesion- and biofilm-associated regulatory pathways in *C. auris*.

In conclusion, our findings demonstrate that BE exerts significant antifungal and anti-virulence activities against *C. auris* by inhibiting growth, suppressing adhesion and biofilm formation, attenuating virulence-associated phenotypes, and regulating adhesion-related transcriptional networks. These results provide new insights into the molecular mechanisms underlying *C. auris* colonization and identify BE as a promising candidate for the prevention and control of *C. auris* infections and transmission.

## MATERIALS AND METHODS

### *C. auris* strains and cultivation

The *C. auris* strains used in this study are all listed in Table 1. Strains BJCA001, FDSA1, FDSA4, FDSA9, and FDSA30 were kindly provided by Professor Huang Guanghua from Fudan University. *C. auris* (BJCA001, FDSA1, FDSA4, FDSA9, and FDSA30) were cultured on YPD solid medium, and then single colonies were picked and transferred into YPD liquid medium for activation. The fungal suspensions were cultured in a shaking incubator at 30°C, 200 rpm until the OD_600_ value was adjusted to 0.1. Fungal cells were subsequently harvested by centrifugation performed at 825 × *g* for a 5 min duration, after which the supernatant was aspirated and discarded. The resulting cell pellets were resuspended in sterile RPMI-1640 medium to obtain final working concentrations of 2 × 10^3^ or 2 × 10^6^ CFU/mL.

### MIC

According to the CLSI M27 protocol guidelines, the MIC of BE (CAS 491-67-8, HPLC ≥98%; Yuanye, Shanghai, China) against *C. auris* strains (BJCA001, FDSA1, FDSA4, FDSA9, and FDSA30) was determined by the microdilution method in a 96-well plate. The fungal suspension was adjusted to a concentration of 2 × 10^3^ CFU/mL, and drugs were serially diluted by a factor of 2. The concentration range of BE was 0.0625–8 μg/mL, and that of FLZ (CAS 86386-73-4; Yuanye, Shanghai, China) was 32–1,024 μg/mL. In each well, 100 μL of the fungal suspension and 100 μL of the drug solution were added, and the plate was incubated at 37°C for 24 h. The MIC of the drugs against *C. auris* was determined by visual inspection of the absence of fungal growth, supplemented by OD_600_ measurement (37).

### MFC

The fungal suspension was adjusted to a concentration of 2 × 10^3^ CFU/mL. Subsequently, the drugs were serially diluted by a factor of 2. The concentration range of BE was 0.0625–8 μg/mL. In each well, 100 μL of the fungal suspension and 100 μL of the drug solution were added. The plate was incubated at 30°C for 24 h. After incubation, 100 μL was taken from each well and spread evenly on YPD solid medium. After 24 h of incubation, the growth of colonies on the plates was observed. If no colonies were observed, the result was recorded as the MFC (21).

### SMIC

The fungal suspension was adjusted to 2 × 10^6^ CFU/mL, and the drug solution was prepared by serial twofold dilution. The final concentration range of BE was 1–32 μg/mL. In each well, 100 μL of the fungal suspension and 100 μL of the drug solution were added and incubated at 30°C for 24 h. After incubation, the supernatant was discarded, and the planktonic cells were washed away with PBS. Then, 50 μL of XTT and menadione (MackLin, Shanghai, China) were added to each well and incubated in the dark for 2 h. The absorbance of each well was measured at 450 nm using a microplate reader. The drug concentration with the most significant reduction in OD_450_ value compared to the drug-free control group was defined as the SMIC (21). The specific grouping for subsequent experiments was shown in Table 2.

### Growth and stress sensitivity spot assay

The concentrations of BJCA001, FDSA1, FDSA4, FDSA9, and FDSA30 were adjusted to 2 × 10^6^, 2 × 10^5^, 2 × 10^4^, 2 × 10^3^, and 2 × 10^2^ CFU/mL. Then, 5 μL of the fungal suspension was spotted onto YPD plates containing calcium chloride (700 mM), BE (8 μg/mL), sodium chloride (2 M), caspofungin (2 μg/mL), hydrogen peroxide (8 mM), fluconazole (32 μg/mL), and amphotericin B (2 μg/mL). The plates were incubated at 30°C for 24 h, followed by digital imaging for documentation (37).

### Time growth curve

The concentration of *C. auris* was adjusted to OD_600_ = 0.1, and the specific grouping (control, BE-H, FLZ) was shown in Table 2. The cultures were grown at 30°C with shaking at 200 rpm. Samples were taken, and the OD_600_ values were measured and recorded at 0, 6, 12, 18, and 24 h (37).

### Flocculation assay

The concentration of *C. auris* was adjusted to 2 × 10^6^ CFU/mL, and the specific grouping was shown in Table 2. After incubation at 30°C for 24 h, the flocculation state of each group was photographed and recorded, and the OD_600_ value was detected (7). Subsequently, the fungal suspension of each group was added to the confocal dish and observed through the DMi8 microscope (Leica, Germany).

### Cell surface hydrophobicity assay

The concentration of *C. auris* was adjusted to OD_600_ = 0.4 for the initial OD_600_ reading (A0). A total of 1.2 mL of each group of BE-fungal suspension mixture was transferred to an EP tube, and then 200 μL of hexadecane was added. It was left at room temperature for 10 min, followed by vortexing at maximum speed for 1 min to mix the hydrocarbon and aqueous phases. After being left at room temperature for another 15 min to facilitate the separation of the two phases, a portion of the lower aqueous phase was taken for the final OD_600_ reading (A1). The proportion of isolated cells was calculated by dividing A1 by A0 (28). Cell surface hydrophobicity (CSH) was calculated using the following formula: CSH = [1 − (A1 / A0)] × 100%.

### Protease assay

Bovine serum albumin (BSA) medium containing 0.05% magnesium sulfate, 2% glucose, 0.1% potassium dihydrogen phosphate, 1% BSA, and 2% agar was prepared. The concentration of *C. auris* was adjusted to 2 × 10^6^ CFU/mL. The specific groups are shown in Table 2. Ten microliters of the BE-fungal suspension mixture was dropped onto the surface of the BSA medium and incubated at 30°C for 3–4 days. The effect of BE on the protease activity of *C. auris* was analyzed based on the zone of precipitation (PZ) value (21). The calculation method of the PZ value is the diameter of the colony divided by the sum of the diameter of the precipitation circle and the diameter of the colony. When the PZ value is 1.0, it indicates no protease activity, and no precipitation circle is observed around the colony. When the PZ value is less than 1.0, it indicates that a precipitation circle appears around the colony, and the larger the precipitation circle, the higher the protease activity of *C. auris*.

### Phospholipase assay

Egg yolk agar medium (Hopebio, Qingdao, China) containing 10% sterile egg liquid was prepared. The concentration of *C. auris* was adjusted to 2 × 10^6^ CFU/mL. The specific grouping was shown in Table 2. Ten microliters of the BE-fungal suspension mixture was dropped onto the surface of the egg yolk agar medium and incubated at 30°C for 2–4 days. The effect of BE on phospholipase secretion is evaluated based on the PZ value (21).

### Esterase assay

Lipase culture medium containing 1% peptone, 0.055% calcium chloride, 5% sodium chloride, 1.5% agar, and 0.5% Tween-80 was prepared. The concentration of *C. auris* was adjusted to 2 × 10^6^ CFU/mL. The specific grouping was shown in Table 2. Ten microliters of the BE-fungal suspension mixture was dropped onto the surface of the lipase culture medium and incubated at 30°C for 3–4 days. The effect of BE on esterase secretion was evaluated based on the PZ value (21).

### Hemolysin assay

The concentration of *C. auris* was adjusted to 2 × 10^6^ CFU/mL. The specific grouping was shown in Table 2. Ten microliters of the BE-fungal suspension mixture was dropped onto the surface of Columbia CNA blood agar plates (HuanKai, Guangdong, China) and incubated at 30°C for 3–4 days. The effect of BE on hemolysin secretion was analyzed by the zone of hemolytic activity (HZ) value. The calculation method of the HZ value is the diameter of the colony divided by the sum of the diameter of the hemolytic activity zone and the diameter of the colony (21). An HZ value of 1.0 indicates no hemolytic activity, and no hemolytic activity zone was observed around the colony at this time, whereas an HZ value less than 1.0 indicates the presence of a hemolytic activity zone around the colony. The smaller the value, the larger the hemolytic activity zone, which indicates that *C. auris* has stronger hemolysin secretion activity.

### *Galleria mellonella* infection model

For each group, 10 *G. mellonella* (Jiacheng, Guilin, China) were randomly selected, and 10 μL of *C. auris* with a concentration of 7.5 × 10^6^ CFU/mL was injected into the right posterior paraventral foot. After 2 h, 10 μL of PBS, BE (8, 16, and 32 μg/mL), and FLZ (32 μg/mL) were respectively injected into the left posterior paraventral feet. The treated larvae were then cultured in a dark environment at 37°C for 5 days, and the number of deaths was recorded every day (21).

### *In vitro* murine ear adhesion assay

The ears of C57BL/6 mice (Ziyuan, Hangzhou, China; Qualification Certificate: SCXK2019-004) of equal size were trimmed clean and washed with PBS. The concentration of *C. auris* was adjusted to 2 × 10^6^ CFU/mL. The specific grouping was shown in Table 2. Each ear was incubated with the BE-fungal suspension mixture at 37°C and 80 rpm for 2 h. After incubation, the ears were washed one to two times with PBS to remove non-adherent cells and then either cultured on YPD plates for photography or subjected to plating experiments to quantify the fungal load (10).

### Artificial skin adhesion assay

The concentration of *C. auris* was adjusted to 2 × 10^6^ CFU/mL. The specific grouping was shown in Table 2; 1.5 mL of the BE-fungal suspension mixture was dropped onto the surface of the artificial skin and evenly spread with a sterile cotton swab. It was then incubated at 37°C for 2 h. After the incubation, the artificial skin was washed one to two times with PBS to remove the non-adherent cells, and then the plate coating assay was conducted to quantify the fungal load (10).

### Cell adhesion assay

In each well of a six-well plate, 2 mL of HaCaT (or A431, AT2, and Caco-2) cells with a concentration of 5 × 10^5^ cells/mL were added and cultured in an environment of 37°C and 5% CO_2_ for 24 h. Then, the concentration of *C. auris* was adjusted to 2 × 10^6^ CFU/mL, and the groups were divided as shown in Table 2. The BE-fungal suspension mixture of each group was added to the six-well plates. The plates were placed in a 37°C and 5% CO_2_ incubator and cultured for another 4 h. After that, the cells were rinsed with PBS two to three times and treated with 4% paraformaldehyde at room temperature for 20 min. Then, the cells were rinsed with PBS again and stained with an enhanced Gram staining kit (21). Finally, the adhesion of *C. auris* was observed under an ECLIPSE Ni microscope (Nikon, Japan).

### Non-biological materials adhesion assay

The concentration of *C. auris* was adjusted to 2 × 10^6^ CFU/mL. The specific groups are shown in Table 2. A total of 1.5 mL of the BE-fungal suspension mixture was dropped onto the surface of medical supplies (mask, polypropylene PP; isolation gown, polypropylene non-woven fabric; gloves, nitrile; infusion tube, PVC; endotracheal tube, silicone; urinary catheter, latex) and evenly spread with a sterile cotton swab. The supplies were then incubated at 30°C for 2 h. After the incubation, the non-biological materials were washed with PBS one to two times to remove unattached cells, and *C. auris* was recovered from the surface of the materials for plate coating experiments to quantify the fungal load (5).

### Biofilm metabolic activity assay

The metabolic activity of *C. auris* was detected by the XTT method. The specific steps were as described in the previous SMIC (21).

### Biofilm crystal violet assay

The concentration of *C. auris* was adjusted to 2 × 10^6^ CFU/mL. The specific grouping was shown in Table 2. After incubation at 30°C for 24 h, the samples were rinsed once with PBS, then stained with 0.5% crystal violet staining solution for 30 min, rinsed two to three times with PBS, and finally the OD_590_ value was measured using a microplate reader (21).

### Porcine skin biofilm model

Porcine skin (obtained from a local slaughterhouse) was repeatedly washed with PBS and 75% ethanol, and then the hair was removed completely. Skin samples 12 mm thick were collected using a skin sampler and placed in 12-well plates, each containing 3 mL of DMEM medium. After culturing for 6 h at 37°C and 5% CO_2_, the samples were rinsed with PBS and thoroughly dried, and then placed on DMEM medium (1 mL of paraffin barrier was added at the junction of the DMEM medium and the porcine skin). The concentration of *C. auris* was adjusted to 2 × 10^6^ CFU/mL, and the specific groups are shown in Table 2. Twenty microliters was evenly applied to the surface of the porcine skin and cultured for another 24 h at 37°C, 5% CO_2_ and without humidity. After the culture was completed, the samples were immersed in pre-cooled 2.5% glutaraldehyde for at least 2 h, and then dehydrated with 25%, 75%, 95%, and 100% ethanol in sequence, each for 10 min. After thorough drying at room temperature, the samples were gold-coated and observed and photographed using SEM (21).

### Transcriptomic sequencing

For transcriptome profiling, total RNA was extracted from *Candida auris* isolates harvested from the untreated control group and BE intervention group, with three independent biological replicates established for each experimental group (*n* = 3). The concentration, purity, and integrity of the extracted RNA were evaluated using the Nanodrop 2000 system and agarose gel electrophoresis. Magnetic beads with oligo(dT) were used to pair with poly(A) through A-T base pairing to separate mRNA from total RNA. Subsequently, this mRNA was added to the fragmentation buffer to obtain small fragments of approximately 300 base pairs and was reverse transcribed into cDNA. After forming a stable double-stranded structure, adapters were ligated, and the products were subjected to fragment screening and library enrichment. Sequencing was performed using the Illumina NovaSeq X Plus sequencing platform. Using the DESeq2 software, genes with |log2FC| > 1 and adjusted *P* value <0.05 were selected for further analysis. Sample relationship analysis, gene set enrichment analysis, and gene enrichment analysis were used to interpret the gene expression data.

### Statistical analysis

All statistical analyses in this study were performed using IBM SPSS Statistics version 26.0 (IBM Corp., Armonk, NY, USA), and all visualization figures were generated using GraphPad Prism 9.5 (GraphPad Software, San Diego, CA, USA). Each independent experiment was carried out with three biological replicates to ensure the reliability and reproducibility of the results. Normally distributed quantitative measurement data were presented as the mean ± SD. For comparisons of quantitative variables among three or more groups, one-way analysis of variance was adopted as the primary statistical method. Post hoc pairwise comparisons were performed using the LSD test for data with equal variance, while the Games-Howell test was applied for data with unequal variance. Statistical significance was defined as a two-sided *P* value <0.05 for all analyses.

## Data Availability

Data supporting the findings are available from the corresponding author upon reasonable request. The transcriptome data has been uploaded to The National Center for Biotechnology Information (PRJNA1431181).
